# Navigating the Complexities of Pemphigus Vulgaris: A Comprehensive Iranian Study

**DOI:** 10.1002/iid3.70317

**Published:** 2026-02-04

**Authors:** Delaram Moosavi, Seyed Mohammad Mahdi Khadem, Afsaneh Sadeghzadeh Bazargan, Kambiz Kamyab Hesari, Mehrnaz Azh, Hamed Zarei Sharif, Nasrin Shayanfar, Azadeh Goodarzi

**Affiliations:** ^1^ Minimally Invasive Surgery Research Center Iran University of Medical Sciences Tehran Iran; ^2^ Department of Dermatology, Rasoul Akram Hospital Iran University of Medical Sciences Tehran Iran; ^3^ Department of Dermatopathology, Raazi Hospital Tehran University of Medical Sciences Tehran Iran; ^4^ Faculty of Medicine Iran University of Medical Sciences Tehran Iran; ^5^ School of Medicine Shahid Beheshti University of Medical Sciences Tehran Iran; ^6^ Department of Pathology, Hazret‐e‐Rasoul Hospital Iran University of Medical Sciences Tehran Iran

**Keywords:** mucosa, pemphigus vulgaris, prognosis, recurrence, treatment

## Abstract

**Introduction:**

Pemphigus vulgaris (PV) is a rare, severe autoimmune disorder characterized by the production of autoantibodies that cause blistering of the skin and mucous membranes, often presenting with oral lesions in 50%–70% of cases. It has a global incidence of 0.5–3.2 per 100,000 people, with variations across regions, and in Iran, the rate is about 1 per 100,000 annually. PV affects both sexes equally and typically manifests in the sixth decade of life, though the age of onset varies internationally, tending to be younger in India and Western countries.

**Methodology:**

In this cross‐sectional study, data were collected from 63 patients diagnosed with PV via telephone interviews. This project was approved by the Research Ethics Committee of Iran University of Medical Sciences. Statistical analyses were performed using SPSS software, version 22.0 (IBM Corp., Armonk, NY, USA).

**Results:**

Among 63 PV patients, 56% were female, and 44% were male, with an average age of 50.17 years and a mean age of onset of 44.91 years (SD = 14.77). Most patients (70%) initially presented with mucosal symptoms, and the average time to diagnosis was approximately 17 months. Common misdiagnoses included aphthous ulcers, lichen planus, and allergic reactions. After diagnosis, most patients (82%) received multiple medications. The most frequently used medications were prednisolone (50 patients, 84.75%), methylprednisolone (10 patients, 16.9%), and rituximab (34 patients, 57.63%).

**Discussion:**

PV in this cohort most often began with mucosal symptoms and was frequently preceded by consultations with non‐dermatology clinicians, contributing to diagnostic delays. Such delays may negatively affect.

## Graphical abstract

We conducted a cross‐sectional analysis of 63 patients diagnosed with pemphigus vulgaris, confirmed by biopsy. Information was collected via telephone interviews. The research aimed to assess the demographic characteristics, initial symptoms, diagnosis delays, and treatment outcomes of the participants.

## 1 Introduction

Pemphigus encompasses a group of rare autoimmune diseases that cause blister formation due to immune‐mediated mechanisms. It mainly includes pemphigus vulgaris (PV), characterized by painful oral and mucocutaneous lesions, and pemphigus foliaceus, which affects only the skin [1]. PV primarily involves the skin and mucous membranes, leading to fragile blisters and erosions. The disease is characterized by the production of IgG autoantibodies against desmogleins 1 and 3, which are essential proteins for cell adhesion within the epidermis [2]. The binding of these autoantibodies triggers the breakdown of intercellular connections, a process known as acantholysis, resulting in blister formation [3]. A notable feature of PV is its oral involvement, which occurs in approximately 80% of patients and often begins as non‐specific erosive lesions rather than intact blisters [4]. These early, nonspecific manifestations frequently delay diagnosis, thereby negatively affecting treatment response and prognosis [5].

The global incidence of PV is estimated to be around 2.83 cases per million people annually, with considerable regional variation [6, 7]. In Iran, previous studies have reported an annual incidence rate of approximately 1 per 100,000, and in a recent study by Zahed et al., the incidence in Fars Province was about 2.7 per million people [8, 9]. The disease is more prevalent among women and typically appears in the sixth decade of life, although the age of onset varies by geographic region [10, 11]. Considering the regional variability in disease onset, incidence, and sex distribution [12], a comprehensive analysis of the Iranian population is essential to provide updated, region‐specific epidemiological data for improving disease management strategies.

Furthermore, studies indicate that oral lesions, particularly recurrent ones, may represent the earliest signs of PV, often leading to misdiagnosis and treatment delays, which can adversely influence prognosis [5, 13, 14]. Additionally, a significant proportion of patients with PV, especially those presenting with oral manifestations, initially consult non‐dermatologic specialists, such as dentists, general practitioners, or otolaryngologists. This frequently results in misdiagnoses, including erythema multiforme (EM), viral infections, or other mucocutaneous conditions, which can further delay appropriate management and worsen outcomes [15, 16, 17, 18].

This study aims to provide updated epidemiological insights into PV in Iran, with a particular focus on the impact of diagnostic delays on treatment outcomes and prognosis. Given that a large proportion of patients first consult non‐dermatology specialists, especially when presenting with mucosal symptoms, this research also seeks to raise awareness among healthcare providers and the public regarding the importance of early dermatologic consultation for persistent mucosal lesions.

## 2 Methodology

This retrospective study was conducted in the pathology laboratories of Rasoul Akram Hospital and Esfand Laboratory, located in Tehran. The study population consisted of all patients diagnosed with PV who were referred to the pathology laboratories of Rasoul Akram Hospital and Esfand Laboratory between 2011 and 2021. The patients with a confirmed diagnosis of pemphigus vulgaris based on lesion biopsy with DIF and histopathological examination, further confirmed by ELISA, regardless of age and gender, were included in this study. Data were collected using a questionnaire designed by the study's lead researchers. The questionnaire was administered through telephone interviews with patients having a confirmed diagnosis of PV. The questionnaire included 20 items, covering occupation, education, age, gender, place of residence, onset of the first oral symptom, diagnosis time by a dermatologist, prior consultations before seeing a dermatologist, diagnoses made before the final diagnosis, treatments prescribed by other physicians before the final diagnosis, onset of the first non‐oral lesion, previous medical history, family history, medication history, social history, number of relapses, relapse severity (mild/moderate/severe), time to achieve full disease control relative to the onset of each patient's disease, and treatments prescribed by dermatologists after the final diagnosis.

Relapse was defined as a patient‐reported recurrence or worsening of mucocutaneous lesions that required escalation of therapy, including an increase in systemic corticosteroid dosage or the addition of a steroid‐sparing agent.

The relapse severity was defined mild as disease controlled with topical therapy and/or ≤ 10 mg/day prednisone equivalent without systemic escalation; moderate as requiring systemic corticosteroids up to ~1.0 mg/kg/day and/or addition of a steroid‐sparing agent without hospitalization; and severe as requiring ≥ 1.0–1.5 mg/kg/day systemic corticosteroids and/or hospitalization or rescue therapy (e.g., rituximab or IVIG) [19, 20].

The questionnaire underwent content and face validation by an expert panel (two dermatologists, one pathologist, one epidemiologist) and was piloted for clarity in a small sample; minor wording adjustments were made.

### 2.1 Ethical Considerations

The research protocol was approved by the Ethics Committee of Iran University of Medical Sciences under the code IR.IUMS.FMD.REC.1400.241 (date: June 26, 2021). Informed consent was obtained from all participants during the telephone contact, and participation in the study was voluntary. Additionally, the confidentiality of participants' identities was assured throughout the study.

### 2.2 Data Analysis

Data were analyzed using descriptive statistics, including means, standard deviations, frequencies, and percentages. For hypothesis testing involving normally distributed variables, parametric tests such as independent *t*‐tests, analysis of variance (ANOVA), and Pearson correlation were applied. For non‐normally distributed variables, non‐parametric tests including the Mann–Whitney U test, Kruskal–Wallis test, and Spearman correlation were used. A significance level of *α* = 0.05 was considered. All analyses were performed using SPSS software, version 22.0 (IBM Corp., Armonk, NY, USA).

## 3 Results

### 3.1 Demographic Data

The study included 63 patients, comprising 35 women (55.56%) and 28 men (44.44%), with a mean age of 50.17 years (SD = 14.40) and a mean age of disease onset of 44.91 years (SD = 14.77). Age distribution showed that 22 patients (34.92%) were between 20 and 40 years, 28 patients (44.44%) were between 41 and 60 years, and 13 patients (20.63%) were over 60 years old. The mean age among female patients was slightly higher at 50.45 years (SD = 14.73) compared to 49.82 years (SD = 14.20) among male patients. Baseline characteristics are presented in Table 1.

**Table 1:** Baseline characteristics.

Total number of patients	63
Age, mean (SD)	50.17 (14.4)
Gender (female/male), *N* (%)	35/28 (55.56/44.4)
Age of onset, mean (SD)	44.91 (14.8)
Mucosal manifestation, *N* (%)	44 (69.8)
Cutaneous manifestation, *N* (%)	19 (30.2)
Differential diagnosis, *N* (%)	Aphthous ulcers (20 (31.7))
	Lichen planus (6 (9.5))
	Allergic reactions (6 (9.5))
Post‐diagnosis treatments, *N* (%)	Prednisolone (50 (84.7))
	Rituximab (34 (57.6))
	Methylprednisolone (10 (16.9))
Recurrence, *N* (%)	31 (49.2)
Time to disease control, month (mean (SD))	5.02 (7.2)

### 3.2 Lesion Characteristics

**Figure 1:**
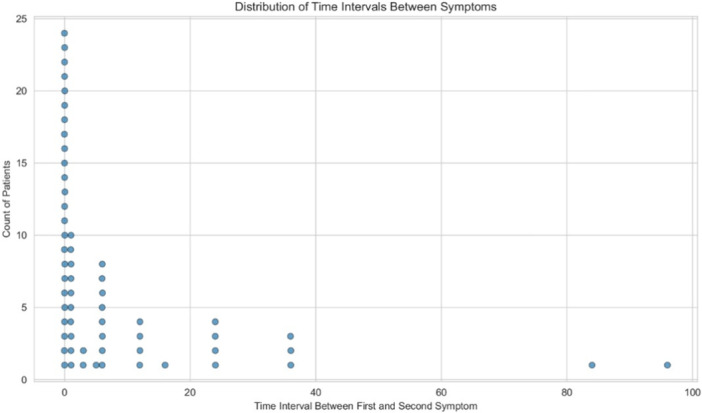
Distribution of the interval (in months) between the onset of the first and second symptoms among participants b. Box plot of time intervals between the first and second symptom (*N* = 59, mean = 8.76, SD = 18.15).

Disease manifestation was predominantly mucosal in 44 patients (69.84%) and cutaneous in 19 patients (30.16%). Throughout their disease course, 24 patients (38%) exclusively had either mucosal (10 patients; 15.87%) or cutaneous symptoms (14 patients; 22.22%). Conversely, 39 patients (62%) experienced both mucosal and cutaneous symptoms (Figure 1). The average time from the onset of the first symptom to definitive diagnosis was 16.69 months (SD = 39.64, range: 1–240 months), with a quicker diagnosis (under 6 months) for 43 patients (69.35%).

### 3.3 Prior Consultations

Before receiving a final diagnosis from a dermatologist, 55 patients (87.30%) had consulted other specialists, while 8 patients (12.70%) were diagnosed on their first visit to a dermatologist. Most pre‐diagnosis consultations were with dermatologists, followed by general practitioners and ENT specialists. Additionally, patients consulted dentists, rheumatologists, infectious disease specialists, and internists. Of these, 19 patients (30.20%) saw general practitioners, 14 patients (22.20%) consulted ENT specialists, 13 visited internists (20.63%), eight went to dentists (12.70%), six saw infectious disease specialists (9.50%), five consulted rheumatologists (7.90%), and three visited other specialists such as gynecologists and oncologists (4.70%).

### 3.4 Differential Diagnoses

In this study, based on the patients' statements, 18 patients (32.14%) received no diagnosis, and 38 patients (67.86%) were diagnosed with conditions other than PV. The most frequently considered differential diagnoses were aphthous ulcers, lichen planus, and allergic reactions, respectively. For 20 patients (31.70%), the initial diagnosis was aphthous ulcers based on the clinical appearance of their lesions. For six patients (9.50%), the initial diagnosis was lichen planus, and for another six patients (9.50%), the initial diagnosis was an allergic reaction. Other diagnoses, such as Behçet's disease, fungal infections, GERD, oral candidiasis, herpes (herpes simplex), and psoriasis, were also considered.

### 3.5 Pre‐Diagnosis Treatments

Based on the initial differential diagnoses, patients received a variety of treatments prior to the final diagnosis of PV. In total, 24 patients (40.70%) did not receive any treatment, while 35 patients (59.30%) were treated; among them, 21 patients (35.59%) received more than two medications. The treatment modalities were categorized into mouthwashes, antibiotics, antifungals, and corticosteroids. Chlorhexidine mouthwash was the most common treatment, prescribed to 16 patients (27.10%), of whom four (25%) received it as their sole treatment. Corticosteroids were given to 14 patients (23.70%), with four (28.57%) receiving them exclusively. Antifungal treatments were administered to eight patients (13.6%), with three (37.50%) receiving only this form of treatment. Lastly, antibiotics were provided to seven patients (11.90%), with three (42.86%) receiving them as their only treatment. This diversity in pre‐diagnosis treatment reflects the challenges in initially diagnosing PV.

### 3.6 Post‐Diagnosis Treatments

Of the 63 patients, four patients were unable to recall the medications they had received after the final diagnosis. Among the remaining 59 patients, one patient (1.70%) did not use any medication post‐diagnosis, 49 patients (83.05%) received multiple medications, and 9 patients (15.25%) received only a single medication. Prednisolone was the most commonly administered medication post‐diagnosis, received by 50 patients (84.75%). Other treatments included Methylprednisolone (10 patients, 16.90%), mycophenolate mofetil (4 patients, 6.80%), Rituximab (34 patients, 57.63%), Azaram (12 patients, 20.34%) Azathioprine (1 patients, 1.70%), Methotrexate (3 patients, 5.08%), Plasmapheresis (1 male patient, 1.70%), Dapsone (1 female patient, 1.70%), Alendronate (2 patients, 3.40%), and Hydroxychloroquine (1 female patient, 1.70%). This highlights the diverse therapeutic approaches adopted post‐diagnosis to manage this autoimmune condition effectively (Table 2).

**Table 2:** The distribution of treatments administered to patients after final diagnosis (%).

Therapy	*N* (%)
Prednisolone	50 (84.75)
Methylprednisolone	10 (16.90)
Rituximab	34 (57.63)
Mycophenolate mofetil	4 (6.80)
Azaram	12 (20.34)
Azathioprine	1 (1.70)
Methotrexate	3 (5.08)
Plasmapheresis	1 (1.70)
Dapsone	1 (1.70)
Alendronate	2 (3.40)
Hydroxychloroquine	1 (1.70)

*Note:* Percentages may exceed 100% because patients often received more than one medication concurrently; therefore, each treatment category is reported independently.

### 3.7 Recurrence

Among the 63 patients, 31 (49.21%) patients reported disease recurrence, whereas 32 (50.79%) patients had no recurrence after diagnosis. Among those who experienced recurrences, the mean number of recurrences was three (SD = 2.80). The average duration between the last two recurrences was 5 months (SD = 9.03). In patients who reported a recurrence of the disease, 17 patients (56.67%) described the severity of the recurrence as mild, 7 patients (23.33%) as moderate, and 6 patients (20%) as severe.

### 3.8 Disease Control and Treatment Response

The average time to disease control was 5.02 months (SD = 7.16), with 91.8% achieving control within 12 months. The mean duration to respond to treatment, reaching a prednisolone dose of 7.5 mg per day, was 8.9 months (SD = 11.75), with no significant gender correlation in recurrence severity (*p* value = 0.93).

Among 63 patients, 56 reported the time to respond to treatment from the onset of the disease and the time to achieve a maintenance dosage of 7.5 milligrams. The mean response time to treatment was 8.9 months, with a standard deviation of 11.75 months. Specifically, 57.14% of the patients (*n* = 32) responded to treatment within 6 months, 32.14% (*n* = 18) within 7–12 months, and 10.71% (*n* = 6) within 12 months or more.

We also found a significant correlation between the number of relapses and their severity (*p* < 0.0001), indicating that more relapses are associated with greater severity.

Additionally, significant correlations were found between the number of relapses and the duration of disease control post‐diagnosis (*p* = 0.003) and between the severity of relapses and the duration of disease control (*p* = 0.027). The interval between disease onset and definitive diagnosis and the number of relapses showed a significant direct correlation (*p* = 0.031), suggesting that earlier diagnosis could lead to fewer relapses. Similarly, this interval was associated with disease control and treatment response, with later diagnosis leading to delayed disease control and a longer time to reduce the Prednisolone dosage to 7.5 mg (*p* values of 0.029 and < 0.0001, respectively).

## 4 Discussion

In this study, the female‐to‐male ratio for pemphigus vulgaris patients was 1.25, indicating a higher prevalence in women than in men, consistent with findings from a study in Turkey by Yayli et al., which reported a ratio of 1.41 [21]. Similar gender predispositions were observed in significant studies by Huang et al. and Chams‐Davatchi et al., with ratios of 1.3 and 1.5, respectively, highlighting the autoimmune disease's tendency to affect women more frequently [22, 23]. This gender bias is further supported by a study conducted by Esmaili et al. in Iran, reporting a ratio of 1.59 [24]. Also, a review study conducted by Kianfar et al. reported a higher occurrence of pemphigus among females, with an average ratio of females to males standing at 1.4:1, varying from 1.1 in Finland to 5.0 in the United States, in accordance with the review study by Rosi‐Schumacher, which reported that women are more influenced than men [25, 26]. But conversely, Zhao et al. reported equal incidence rates among men and women [6].

The average age of diagnosis among the patients in this study was 50.17 ± 14.1 years, aligning closely with previous research indicating no significant gender disparity in diagnosis age (*p* = 0.897). This contrasts with Huang et al., who found slightly higher age averages in their study. The prevalence of Pemphigus Vulgaris in middle‐aged patient groups, especially in the fifth decade, was observed, aligning with the disease's demographic characteristics reported in the broader literature [23]. Furthermore, according to a study conducted by Askin et al., the mean age at diagnosis was reported as 50.4 ± 13.7 years [27]. Based on another study by Porro et al., PV is mostly observed among individuals aged between 40 and 60 years [28]. Moreover, in a review study by Kridin et al., the majority of patients are typically diagnosed between the ages of 45 and 65 [11].

Pemphigus vulgaris primarily manifests as superficial mouth ulcers, particularly affecting the lip and buccal mucosa, though other mucosal surfaces can be involved [29]. Our findings indicate that the disease initially presents with mucosal symptoms in 69.84% of patients and with skin symptoms in 30.16%, which is consistent with Chams‐Davatchi et al.'s observations of a higher prevalence of mucocutaneous involvement [22]. According to Ingold et al., PV typically manifests first within the oral mucosa in 80% of instances. These blisters within the mouth frequently rupture, resulting in painful erosions. Cutaneous lesions tend to develop in approximately 75% of PV patients following the initial oral blistering [12]. According to another study by Wojnarowska et al., the initial presentation involves oral lesions in 50% to 70% of patients, which subsequently develop in 90% of patients during the illness [30].

Interestingly, the average time from the first symptom to definitive diagnosis in our study was significantly shorter in patients whose disease presented with mucosal symptoms compared to those with skin manifestations (13 months vs. 26 months, respectively, *p* = 0.02), a phenomenon also reported by Chams‐Davatchi et al. This delay might be attributed to patients with skin manifestations delaying medical consultation and those with painful mucosal lesions seeking immediate care [22]. Based on the study by Daltaban et al., patients initially presenting with desquamative gingivitis had a significantly longer diagnostic delay time (8.25 months) compared to those with ulcers and erosions (4.78 months) [31]. Also based on Sultan et al., the period from initial presentation to a confirmed diagnosis typically averages 6 months [32]. Other studies indicated that pemphigus is often identified later when it manifests in the oral cavity compared to the skin, owing to the more ambiguous characteristics associated with mucosal involvement [33, 34].

A notable finding from our study is the substantial rate (87.3%) of patients consulting non‐dermatologists prior to reaching a definitive diagnosis, with the majority first visiting dermatologists, general practitioners, and ENT specialists. Ljubojevic et al. have reported that oral pemphigus vulgaris is often misdiagnosed by family physicians or dentists [35]. Similarly, Chams‐Davatchi et al. have noted that in Iran, given the more common occurrence of oral manifestations, patients tend to consult general practitioners and dentists more frequently [36]. It is noticeable that Patients often face challenges including inconclusive tests and sometimes dismissive attitudes from healthcare providers, leading to feelings of resignation. The study sheds light on the patients' criticisms of the medical approach during this taxing diagnostic journey, highlighting the need for more understanding and efficient diagnostic processes in managing rare autoimmune diseases like pemphigus [37]. Also based on the study by Venugopal et al., differential diagnoses for these patients include conditions like herpes, aphthous ulcers, lichen planus, paraneoplastic lesions, and other autoimmune diseases [38]. Oral lesions in PV can resemble various other conditions, such as aphthous stomatitis, acute herpetic stomatitis, erythema multiform, lichen planus, systemic lupus erythematosus, paraneoplastic pemphigus, and mucous membrane pemphigoid, which makes the differential diagnosis extensive. Blisters within the oral cavity tend to be transient, and biopsies of these erosions often fail to confirm the diagnosis [30, 38, 39]. In the case of skin lesions associated with pemphigus vulgaris (PV), the differential diagnosis encompasses various conditions, such as different types of pemphigus, bullous pemphigoid, linear IgA bullous dermatosis, bullous forms of erythema multiforme, and dermatitis herpetiformis [28]. In our study, we observed that most patients were given diagnoses such as aphthous ulcers, lichen planus, allergic reactions, Behçet's disease, fungal infections, GERD, etc. The three most frequently mentioned differential diagnoses were aphthous ulcers, lichen planus, and allergic reactions, in that order.

Regarding treatment, the majority of our patients received a combination therapy of Prednisolone and Rituximab, reflecting current trends toward using immunosuppressants and biological agents for patients with complications or recurrences [40]. Atzmony et al.'s systematic review and meta‐analysis found that azathioprine and cyclophosphamide reduced corticosteroid requirements but did not increase remission rates. Mycophenolate mofetil was associated with quicker remission and delayed relapse without lowering steroid use. Intravenous immunoglobulin provided short‐term benefit, and topical epidermal growth factor was linked to improved lesion healing [41]. Moreover, based on a systematic review and meta‐analysis by Atzmony et al., in which they evaluated different adjuvants such as azathioprine, and mycophenolate mofetil, among others, found that although these adjuvants didn't aid in achieving remission, they collectively decreased the risk of relapse by 29%. This indicates their potential benefit in pemphigus management, highlighting the importance of further research on treatment alternatives [42]. Although, based on the recent study by Abulikemu et al., the main treatments for pemphigus, like systemic corticosteroids, and immunosuppressants, can cause severe side effects. Current and investigational treatments include monoclonal anti‐CD20 antibodies and various specific pathway inhibitors, showing promise in clinical use or trials. Future directions may explore additional targeted options like IL‐4Rα antibody and IL‐17 blockers, offering hope for more effective and safer pemphigus management strategies [43].

This study has several limitations. Its retrospective, telephone‐based design introduces potential recall bias, particularly in patient‐reported data on symptom onset, relapse timing, and severity. The absence of standardized severity scoring (e.g., PDAI) prevented severity‐adjusted analyses. Additionally, treatment regimens varied and were not consistently documented, limiting comparative evaluation. The small sample size, lack of a control group, and single‐center setting further reduce the generalizability of findings.

## 5 Conclusion

It seems that PV is more common in women, particularly those in their middle years, especially during their fifties. This disease often starts with signs in the mucous membranes, making these types of symptoms more common than those on the skin. When the disease begins with skin symptoms, it is often recognized later, which seriously affects recovery and disease management.

Future research on PV should focus on improving early diagnosis, evaluating the long‐term effectiveness of treatments, and developing personalized care approaches tailored to individual patient needs. Implementing patient education tools, including digital health solutions, could significantly enhance disease management and quality of life for those affected by PV.

## 6 Author Contributions

Delaram Moosavi contributed to the study conception and design, data collection, and drafting of the manuscript. Seyed Mohammad Mahdi Khadem contributed to the study design, data analysis, interpretation of the results, and critical revision of the manuscript. Afsaneh Sadeghzadeh and Kamran Bazargan contributed to data collection and patient interviews. Kambiz Kamyab Hesari, Mehrnaz Azh, and Hamed Zarei contributed to clinical data interpretation and manuscript revision. Sharif Shayanfar and Nasrin Azadeh contributed to statistical analysis and interpretation of data. Azadeh Goodarzi supervised the study, contributed to the interpretation of findings, and critically revised the manuscript for important intellectual content. All authors read and approved the final manuscript.

## Funding

The authors received no specific funding for this work.

## Ethics Statement

This study was conducted in accordance with the ethical standards of the ethics committee of Iran university of medical sciences (approval number: IR.IUMS.FMD.REC.1400.241, date: June 26, 2021), and with the 2013 Helsinki declaration and its later amendments or comparable ethical standards.

## Consent

A signed written informed consent was obtained from the patients.

## Conflicts of Interest

The authors declare no conflicts of interest.

## Data Availability

The data sets generated and/or analyzed during the current study are not publicly available due to privacy and ethical restrictions imposed by the Research Ethics Committee of Iran University of Medical Sciences. However, aggregate data supporting the findings of this study are available from the corresponding author upon reasonable request, subject to approval by the Research Ethics Committee. Additional details and requirements for accessing the data can be provided upon direct contact with the research team.
